# PD-L1 expression in renal cell carcinoma clear cell type is related to unfavorable prognosis

**DOI:** 10.1186/s13000-015-0414-x

**Published:** 2015-10-15

**Authors:** Katia R M Leite, Sabrina T. Reis, José Pontes Junior, Marcelo Zerati, Daniel de Oliveira Gomes, Luiz H. Camara-Lopes, Miguel Srougi

**Affiliations:** Department of Urology, Laboratory of Medical Research, University of São Paulo Medical School, Sao Paulo, Brazil; Department of Molecular Biology, Genoa Biotecnologia SA, Av Dr. Arnaldo #455, room 2145, 01246-903 Sao Paulo, Brazil

**Keywords:** Renal cell cancer, PD-L1, Prognosis, Immunotherapy, Immunohistochemistry

## Abstract

**Background:**

PD-L1 is a glycoprotein from the family of T-cell co-stimulatory molecules that are constitutively expressed by macrophages. Aberrant expression of PD-L1 is observed in human cancers associated with inhibition of the tumor-directed T-cell immune response. There are few reports in the literature evaluating PD-L1 expression in association to prognosis specifically in renal cell cancer clear cell type (RCC-CC).

**Methods:**

Immunohistochemistry using a PD-L1 polyclonal antibody was performed on a tissue microarray (TMA) that contained 115 surgical specimens of RCC-CC. Cases were classified based on the absence or presence of staining intensity in the cytoplasm and membranes of the tumor cells. Statistical analysis was used to determine the association of PD-L1 expression with classic prognostic factors and tumor recurrence.

**Results:**

PD-L1 expression was positive in 56.5 % of tumors. The univariate analysis showed a correlation between PD-L1 expression and nuclear Fuhrman grade (*p* = 0.021) and microvascular tumor embolization (*p* = 0.039). One hundred and four patients were monitored for a mean time of 115.7 months. Seventeen patients (16.3 %) suffered tumor recurrence. Negative outcomes were associated with higher nuclear grade tumors, PD-L1 expression, and the presence of microvascular invasion.

**Conclusion:**

Our findings confirm that PD-L1 expression is an important prognostic factor in RCC-CC.

## Background

Recently, the capacity of neoplastic cells to evade immunological destruction became an additional checkpoint in assessing the hallmarks of cancer [1]. T cells play the most important role in this context; the recognition of tumor-associated antigens by healthy T cells allows the activation of a specific anti-tumor immune reaction. CD8+ effector T cells, known as cytotoxic T lymphocytes (CTLs), are the main players in this process. Two receptors, cytotoxic T-lymphocyte-associated antigen 4 (CTLA4) and programmed cell death protein 1 (PD-1), have been actively studied in cancer for their potential roles as inhibitory receptors. Blockage of these receptors by antibodies has been studied in numerous clinical trials with promising results [2, 3].

PD-1 is a 288-amino acid cell-surface protein. PD-1 binds two ligands, PD-L1 and PD-L2, which negatively regulate the immune response. The expression of PD-L1 (also known as B7-H1) on tumor cells leads to the inhibition of the T cell-mediated immune response against cancer, thereby enabling tumor progression and metastasis [4, 5].

Expression of PD-L1 has been correlated with poor clinical outcomes in a number of human cancers [6], including renal cell cancer (RCC) [7]. As a result, it has been considered a potential predictive biomarker and has inspired new drug development designed to block PD-1.

Immunotherapy was the leading strategy for treating RCC until recently, when targeted inhibitors of the VEGF (Vascular endothelial growth factor) and mTOR pathway began to show promising results. These therapeutic inhibitors increased progression-free and overall survival rates, but failed to show a durable response. Blocking the PD-1-PD-L1 interaction with monoclonal antibodies, however, restores the activity of T cells within the tumor microenvironment and has been shown to result in a significant and sustained antitumor response in clinical trials [8].

Our aim is to study the PD-L1 expression in RCC clear cell type (RCC-CC) and how that expression correlates with prognostic factors and tumor recurrence.

## Methods

The Institutional Internal Review Board approved this study (process number 1,034,579). We retrospectively analyzed surgical specimens from 148 patients diagnosed with localized (NX-0 M0) RCC-CC who underwent radical or conservative renal surgery (partial nephrectomy or tumor enucleation) between 1988 and 2006 at our institution. Data and adequate material for examination were available for 115 patients, and the clinical and pathological characteristics are shown in Table 1. For staging purposes, lymph node dissection was limited to the hilar region in those who underwent radical nephrectomy. The same surgeon (MS) operated on all patients and all pathological analyses were performed by the same uropathologist (KRML). Patients with systemic metastatic disease at the time of surgery were excluded from the study. For each patient, the analyzed clinical and pathological characteristics included age, gender, symptoms at initial presentation, tumor size, pT stage (2010 TNM classification), Fuhrman nuclear grade, nucleolar grade as recently recommended by International Society of Urological Pathology (ISUP) [9], coagulative tumor necrosis, and microvascular invasion. Following surgery, all patients appeared for regular follow-up visits based on their staging. Low-risk patients returned for semi-annual physical examinations and routine blood tests in addition to annual chest radiography and abdominal computed tomography. Chest tomography, bone scintigraphy, and brain imaging were conducted in clinically applicable cases.

**Table 1:** Clinical and pathological characteristics of 115 patients with renal cell cancer, clear cell type studied for the presence of PD-L1 immune-expression

	*N *(%)
Age (years old)	
Mean (SD)	57.7(11.5)
Gender	
Male	91(79.1)
Female	24(20.9)
Tumor size (cm)	
Mean (SD)	4.8(2.9)
Tumor stage	
pT1	80(69.6)
pT2	8(7.0)
pT3	27(23.4)
Metastasis to lymph nodes	6(5.2)
Fuhrman nuclear grade	
1	25(21.7)
2	44(38.3)
3	41(35.7)
4	5(4.3)
Nucleolar grade	
1	22(19.1)
2	47(40.9)
3	46(40.0)
Microvascular invasion	
Absent	83(72.2)
Present	32(27.8)
Tumor necrosis	
Absent	91(79.1)
Present	24(20.9)
Presentation	
Incidental	76(66.1)
Symptoms	39(33.9)

The tissue microarray (TMA) was constructed as previously described [10]. Using a precision mechanical system (Beecher Instruments, Sun Prairie, WI), tissue cylinders with a diameter of 0.6 mm were removed from each patient’s paraffin block containing the RCC-CC from specific areas, corresponding to the previously demarcated, most representative areas from respective hematoxylin-eosin-stained slides. These cylinders were transferred with 3-mm intervals to a recipient paraffin block. Next, the tissue microarray recipient block was cut into 3-μm histologic section, and this slide was used for immunohistochemistry. Two tumor samples were collected per patient, because it has been demonstrated that analysis of 2 disks is comparable to the analysis of a whole tissue section in more than 95 % of cases [11].

Immunohistochemical analysis was performed using BOND III Leica equipment (Leica Biosystems) using the polyclonal antibody anti-PD-L1 (ABCAM) in a 1:25 dilution.

Statistical analysis was performed using SPSS version 19.0 software. Differences between groups were evaluated using a *χ*^2^ test. Time to recurrence was calculated using the Kaplan–Meier method. The differences between the curves were measured using a log-rank test. Two-tailed tests were used and a p*-*value <0.05 was considered to be significant.

## Results

**Fig. 1:**
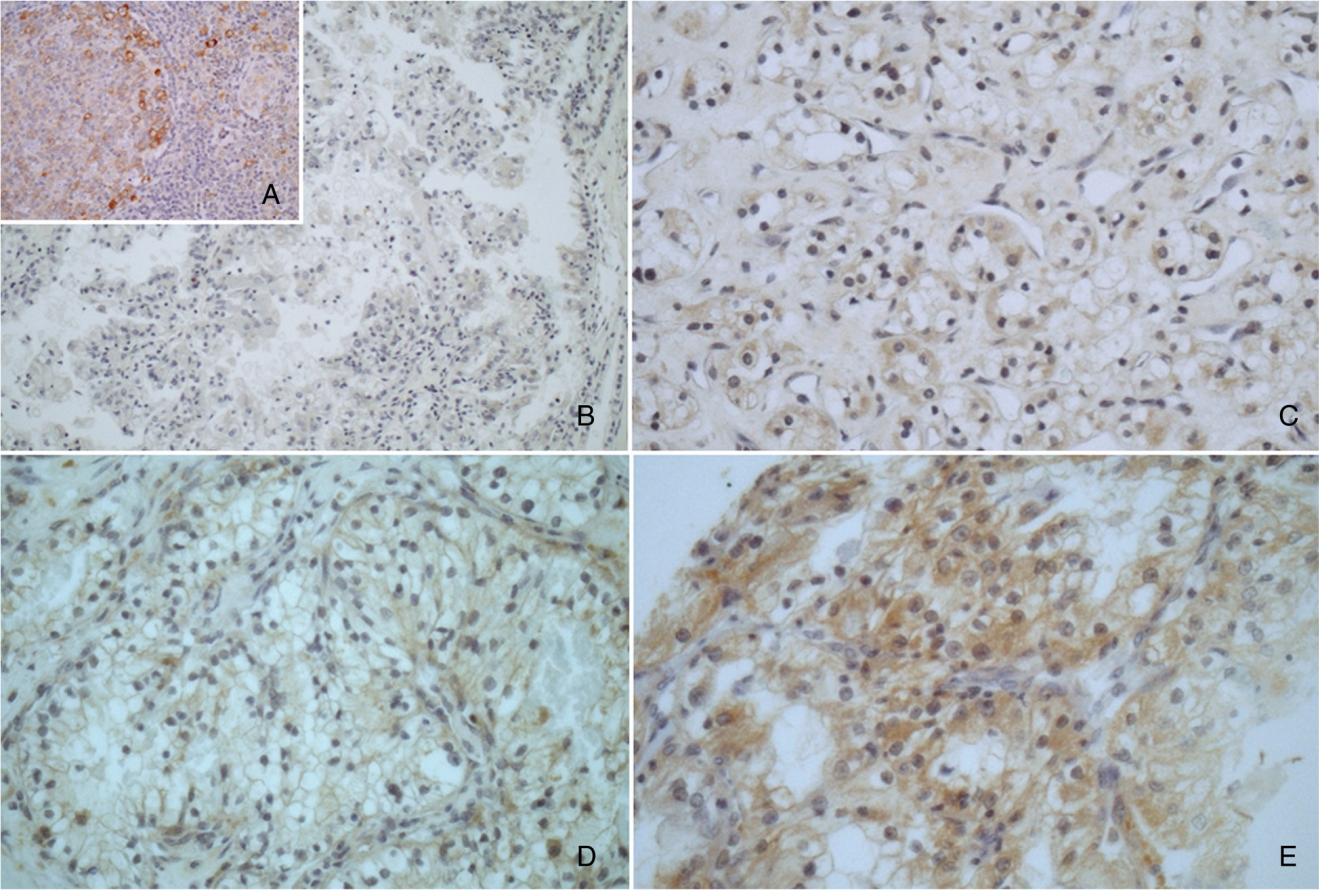
Photomicrography showing PD-L1 immune-expression in RCC-CC. **a** Positive control, **b** negative control, **c** Positive - score 1, **d** Positive – score 2, **e** Positive – score 3

PD-L1 was diffusely expressed in the cytoplasm and membrane of tumor cells in 65 (56.5 %) of assessed cases. The staining was weak in 29 (44.6 %), moderate in 15 (23.1 %) and strong in 21 (32.3 %) of the cases (Fig. 1). For statistical analysis, we considered any intensity of staining as positive and compared these results with cases that were completely negative for staining. The univariate analysis showed correlation between PD-L1 expression and higher nuclear Fuhrman grade (*p* = 0.021) and microvascular tumor embolization (*p* = 0.039). The results are expressed in Table 2. Although there was no correlation between PD-L1 expression and any other of the tested negative prognostic factors, in the few cases in which lymph node metastasis was present, PD-L1 was expressed in 67 % of the cases. PD-L1 was also expressed in 83 % of cases that developed distant metastasis and in 65 % of cases that showed tumor recurrence.

**Table 2:** Correlation between PD-L1 expression and prognostic factors

	PD-L1 neg	PD-L1 pos	*p*
Characteristics (*N* = 115)			
Age	58.2	57.4	0.737
Gender			
Female	9(37.5)	15(62.5)	0.507
Male	41(45.1)	50(54.9)	
Tumor size (cm)	4.5	5.1	0.246
Presentation			
Incidental	34(44.7)	42(55.3)	0.704
Symptomatic	16(41.0)	23(59.0)	
Fuhrman nuclear grade			
1,2	36(52.2)	33(47.8)	**0.021**
3,4	14(30.4)	32(69.6)	
Nucleolar grade			
1	12(54.5)	10(45.5)	0.207
2	16(34.0)	31(66.0)	
3	22(47.8)	24(52.2)	
Microvascular invasion			
Absent	41(49.4)	42(50.6)	**0.039**
Present	9(28.1)	23(71.9)	
Tumor necrosis			
Absent	41(45.1)	50(54.9)	0.507
Present	9(37.5)	15(62.5)	
Tumor stage			
pT1	38(47.5)	42(52.5)	0.188
pT2,3	12(34.3)	23(65.7)	
Metastasis to lymph nodes			
Absent	48(44.0)	61(56.0)	0.607
Present	2(33.3)	4(66.7)	
Development of distant metastasis			
Absent	49(45.0)	60(55.0)	0.174
Present	1(16.7)	5(83.3)	
Tumor recurrence (*N* = 104)			
Absent	39(44.8)	48(55.2)	0.468
Present	6(35.3)	11(64.7)

**Table 3:** Clinical and pathological characteristics of patients considering tumor recurrence

Tumor recurrence (*N* = 104)	No(87)	Yes(17)	*p*
Characteristics			
Age (mean-yo)	57.7	60.1	0.436
Gender			
Female	21(24.1)	3(17.6)	0.561
Male	66(75.9)	14(82.4)	
Tumor size (mean-cm)	4.1	8.6	<0.001
Presentation			
Incidental	64(73.6)	4(23.5)	<0.001
Symptomatic	23(26.4)	13(76.5)	
Fuhrman nuclear grade			
1,2	57(65.5)	8(47.1)	0.150
3,4	30(34.5)	9(52.9)	
Nucleolar grade			
1	17(19.5)	2(11.8)	0.743
2	36(41.4)	8(47.1)	
3	24(39.1)	7(41.2)	
Microvascular invasion			
Absent	70(80.5)	5(29.4)	<0.001
Present	17(19.5)	12(70.6)	
Tumor necrosis			
Absent	72(82.8)	9(52.9)	0.007
Present	15(17.2)	8(47.1)	
Tumor stage			
pT1	70(80.5)	5(29.4)	<0.001
pT2,3	17(19.5)	12(70.6)	
Metastasis to lymph nodes			
Absent	86(98.9)	13(76.5)	<0.001
Present	1(1.1)	4(23.5)	
PDL1			
Negative	39(44.8)	6(35.3)	0.468
Positive	48(55.2)	11(64.7)	

One hundred and four patients were followed for a mean time of 115.7 months. Seventeen (16.3 %) of the followed cases suffered tumor recurrence. Tumor size (8.6 cm vs 4.1 cm; *p* < 0.001), symptomatic presentation (*p* < 0.001), microvascular invasion (*p* < 0.001), tumor necrosis (*p* = 0.007), tumor stage (pT2/pT3 vs pT1, *p* < 0.001) and the presence of lymph node metastasis (*p* < 0.001) were all factors that were related to tumor recurrence (Table 3).

**Fig. 2:**
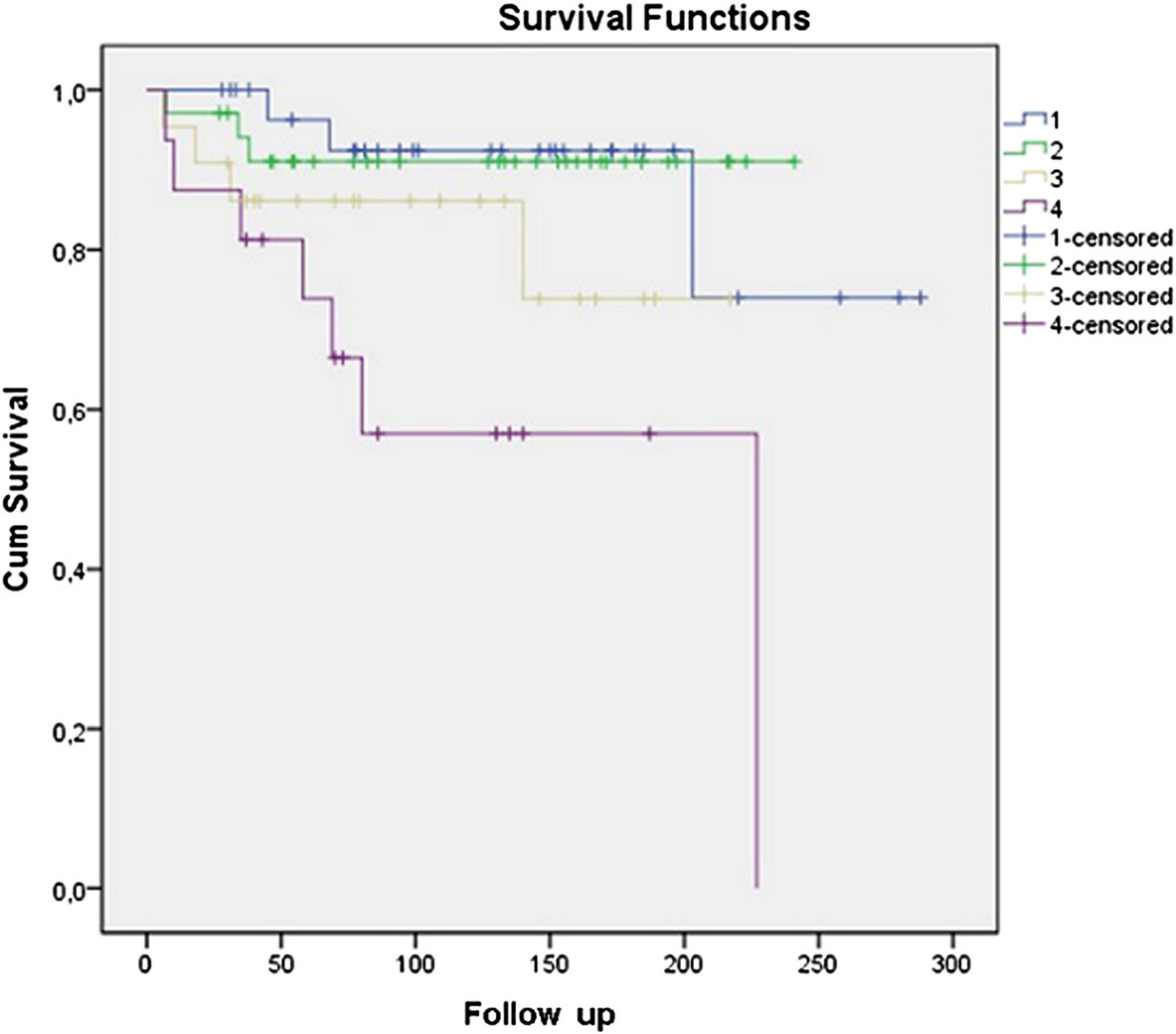
Kaplan-Meier curve of tumor recurrence. The blue line represents tumors negative for PD-L1 with no microvascular tumor invasion and low nuclear grade. The green line represents tumors with one of the following: PD-L1 expression, microvascular tumor invasion, or high nuclear grade. The yellow line represents tumors with two of these variables, and the purple line represents tumors with all three unfavorable prognostic factors (*p* = 0.007)

We have previously described that the Fuhrman nuclear grade and microvascular tumor invasion are powerful predictors of outcome in RCC [12]. Considering these two important prognostic factors for localized RCC, we built a Kaplan-Meier curve using these parameters with the addition of PD-L1 expression. Cases were grouped as follows: 1. PD-L1 negative, no microvascular tumor invasion and low Fuhrman nuclear grade; 2. Presence of one of any of these variables; 3. Presence of two of any of these variables; and 4. Presence of all three variables: PD-L1 expression, microvascular tumor invasion and high Fuhrman nuclear grade. The curves are shown in Fig. 2. There is clearly a higher rate of tumor recurrence in patients who were positive for these three poor prognostic factors (*p* = 0.007).

Fourteen (12.2 %) patients died, 13 (92.9 %) of whom died as a result of RCC progression. PD-L1 was positive in 8 from these 13 (61.5 %) cases (*p* = 0.104). Although the relationship is not statistically significant, the small number of tumor-related deaths may have influenced the negative result.

In conclusion, PD-L1 was diffusely expressed by 56.5 % of the cases related to higher nuclear Fuhrman grade (*p* = 0.021) and microvascular tumor embolization (*p* = 0.039) in univariate analysis.

## Discussion

PD-L1 expression has been studied as a biomarker of response to the new PD-1/PD-L1 inhibitors in different tumors, but its prognostic value is not yet well established.

Our study showed that PD-L1 was expressed in 56.5 % of RCC-CC cases and positive expression was correlated with a higher Fuhrman nuclear grade and microvascular tumor embolization. In addition, PD-L1 analysis provided information about the relationship between tumor outcome and higher rates of tumor recurrence when associated with these two other tested prognostic factors.

In 1999, Dong et al. described PD-L1 as a cell-surface glycoprotein within the B7 family of T-cell co-stimulatory molecules that is constitutively expressed by macrophage-lineage cells [13]. Since then, studies have shown aberrant expression of PD-L1 in various human cancers, including breast, ovarian, lung, and colon cancer, in addition to lymphoma and melanoma [14]. Tumor cells that express PD-L1 have been shown to inhibit tumor-specific T-cell-mediated immunity by inducing T-cell apoptosis, impairing cytokine production, and diminishing the cytotoxicity of activated T cells [15].

There are few reports in the literature evaluating the relationship between PD-L1 expression and clinical prognosis in RCC, and the majority of existing reports relates expression with other known poor prognosis factors and negative outcome.

Thompson et al. were the first to study PD-L1 expression by immunohistochemistry in RCC, finding 24 % of staining associated with adverse pathologic features, including higher tumor stage, greater tumor size, a Fuhrman nuclear grade of 3 or 4, and tumor necrosis [16]. In addition, by evaluating the expression levels of PD-L1 in tumor cells alone, in lymphocytes alone, or in tumor and/or lymphocytes combined, researchers have shown an association between PD-L1 positivity with aggressive tumor behavior and increased risk of death from RCC [17].

Choueiri et al. showed that PD-L1 expression is related to shorter survival times in patients with metastatic RCC who were receiving VEGF-targeted agents, therefore arguing that PD-L1 expression should be considered in the design of future clinical trials [18].

Interestingly a study published by Jilaveanu et al. [19] compared PD-L1 in 34 cases of primary and metastatic RCC showing no correlation between paired specimens. They advocate that the result of primary tumor regarding PD-L1 expression is not appropriate to predict response to the new PD-L1 inhibitors.

In non-clear cell RCC patients with PD-L1 tumor expression is associated with higher tumor stage and grade and worse clinical outcomes [20].

In breast cancer, PD-L1 was upregulated in 20 % of cases and was related to poor-prognostic features such as tumor size, high grade, estrogen and progesterone negativity, Her2/Neu positivity and higher rates of cell proliferation, although upregulation of PD-L1 was not associated with survival of patients in this study [21].

In gastric cancer, PD-L1 was positively expressed in 50 % of cases associated with higher T stage, lymph-node metastasis and overall survival [22].

A meta-analysis study of non-small cell lung cancer indicated that PD-L1 expression is associated with tumor differentiation and lower overall patient survival [23].

The fact that we used a TMA to indicate PD-L1 expression should be a point of criticism; however, in studies of other tumor markers, the representation of two cores has been shown to generally be similar to a whole tissue fragment, with the advantage of standardization in the immunohistochemistry reaction [11]. The small number of cases, particularly the small number of cancer deaths, may reflect some percentage of error in TMA-based PD-L1 analysis, which may explain the absence of a positive result regarding PD-L1 expression and tumor prognosis in this statistical analysis.

## Conclusion

In conclusion, this report confirms, using immunohistochemistry, that PD-L1 expression represents a new marker of prognosis in RCC-CC.

## Abbreviation

RCC-CC: Renal cell cancer clear cell type
CTL: Cytotoxic T lymphocytes
CTLA4: C ytotoxic T-lymphocyte-associated antigen 4
PD-1: Programmed cell death protein 1
PD-L1: Programmed cell death protein ligand 1
VEGF: Vascular endothelial growth factor
mTOR: Mammalian target of rapamycin
TMA: Tissue microarray
